# A Missing Dimension in Measures of Vaccination Impacts

**DOI:** 10.1371/journal.ppat.1003849

**Published:** 2014-03-06

**Authors:** M. Gabriela M. Gomes, Marc Lipsitch, Andrew R. Wargo, Gael Kurath, Carlota Rebelo, Graham F. Medley, Antonio Coutinho

**Affiliations:** 1 Instituto Gulbenkian de Ciência, Oeiras, Portugal; 2 Center for Communicable Disease Dynamics, Harvard School of Public Health, Boston, Massachusetts, United States of America; 3 Virginia Institute of Marine Science, College of William and Mary, Gloucester Point, Virginia, United States of America; 4 U.S. Geological Survey, Western Fisheries Research Center, Seattle, Washington, United States of America; 5 Faculdade de Ciências de Lisboa e Centro de Matemática e Aplicações Fundamentais, Campo Grande, Lisboa, Portugal; 6 School of Life Sciences and WIDER, University of Warwick, Coventry, United Kingdom; The Fox Chase Cancer Center, United States of America

Immunological protection, acquired from either natural infection or vaccination, varies among hosts, reflecting underlying biological variation and affecting population-level protection. Owing to the nature of resistance mechanisms, distributions of susceptibility and protection entangle with pathogen dose in a way that can be decoupled by adequately representing the dose dimension. Any infectious processes must depend in some fashion on dose, and empirical evidence exists for an effect of exposure dose on the probability of transmission to mumps-vaccinated hosts [1], the case-fatality ratio of measles [2], and the probability of infection and, given infection, of symptoms in cholera [3]. Extreme distributions of vaccine protection have been termed leaky (partially protects all hosts) and all-or-nothing (totally protects a proportion of hosts) [4]. These distributions can be distinguished in vaccine field trials from the time dependence of infections [5]. Frailty mixing models have also been proposed to estimate the distribution of protection from time to event data [6], [7], although the results are not comparable across regions unless there is explicit control for baseline transmission [8]. Distributions of host susceptibility and acquired protection can be estimated from dose-response data generated under controlled experimental conditions [9]–[11] and natural settings [12], [13]. These distributions can guide research on mechanisms of protection, as well as enable model validity across the entire range of transmission intensities. We argue for a shift to a dose-dimension paradigm in infectious disease science and community health.

## Natural Transmission

We consider a minimal susceptible (*S*) and infected (*I*) model [14] of pathogen transmission in a host population to explore population effects of protection conferred by a vaccine (or other preventive measure, such as symbionts) against infection, under different assumptions about how this is distributed among individuals. We consider that infection is lifelong, and that there is no naturally acquired immunity.

Vaccines that provide leaky protection against infection act by reducing susceptibility to a factor *σ* that is distributed among individuals according to a probability density function 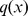, where 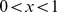. Denoting by 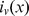 the densities of hosts who are vaccinated and have susceptibility factor 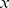, the integral 
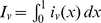
 represents the proportion of hosts who are infected despite being vaccinated. Assuming no effect on infectiousness, the per capita rate of infection among totally susceptible individuals is given by 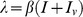, where *β* is the effective contact rate.

**Figure 1:**
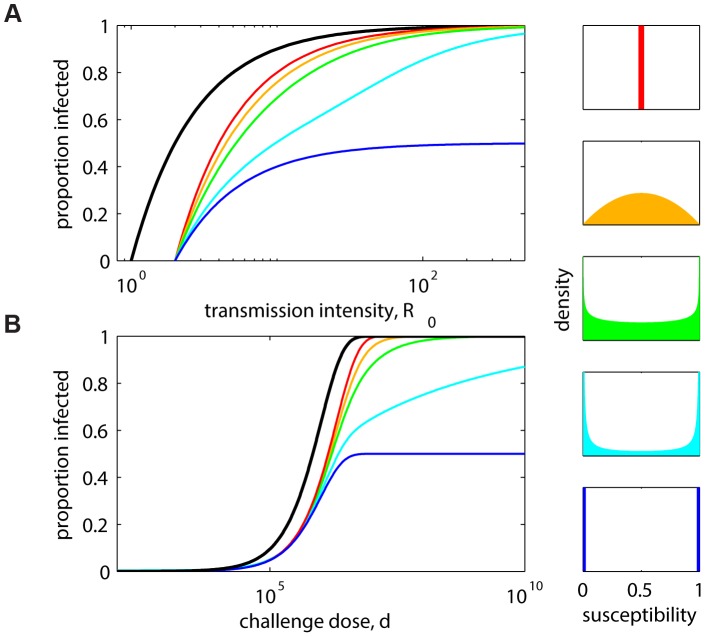
Decreasing infection with heterogeneity in host protection. (A) Equilibrium prevalence of infection under a pathogen transmission model in which an intervention (vaccine or symbiont) reduces host susceptibility to a factor that is distributed as specified. The model is formally represented by the rates of change in the proportions of the population that are susceptible and infected: 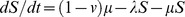, 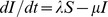, 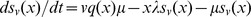, and 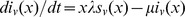, where *S* and *I* are nonintervention, while 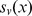 and 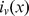 are intervention groups with susceptibility *x* distributed as 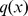 (right panels). Colored lines assume total intervention coverage (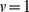), while the black line represents the scenario without intervention (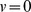). (B) Dose-response curves expected from an experiment in which groups of naive (black) and intervention (colored) hosts are challenged with a range of pathogen doses, under a model in which the intervention reduces susceptibility to a factor that is distributed as in panels on the right. Models for infected proportions in nonintervention and intervention groups are formalized in a dose-response manner by 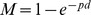 and 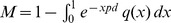, respectively, where *d* is the number of pathogens the host is challenged by and *p* is the probability of infection for each pathogen. Colored lines assume susceptibility factors distributed with mean 0.5 in all cases and variance 0 (red), 0.05 (orange), 0.1 (green), 0.2 (cyan), and 0.25 (blue). Red and blue at the extremes are discrete, while the intermediate cases are continuous beta distributions, with shape parameters *a* and *b* such that the mean is fixed,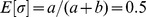, and the variance, 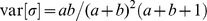, spans the range, 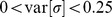. Transmission models assume 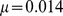, and controlled infection models assume 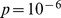.

Figure 1A shows, for the distributions 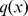 represented on the right, equilibrium curves describing prevalence of infection versus transmission intensity measured by 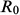, the basic reproduction number. The curves for extreme cases of vaccines that confer equal protection to all or total protection to some and none to others are depicted by the higher (red) and lower (blue) curves, respectively. Intermediate curves represent scenarios in which susceptibility follows a beta distribution with fixed mean and increasing variance from top to bottom. Prevalence curves become shallower with heterogeneity and converge to the same level as transmission increases, except in the all-or-nothing extreme, in which the prevalence cannot surpass the susceptible fraction, irrespective of transmission intensity. Although the impact of protection appears to increase with polarization of effects, the endemic curves do not converge uniformly to their all-or-nothing homologue. In the absence of unequivocal empirical evidence for the idealized all-or-nothing mode of action, we suggest modifying the terminology to include polarized distributions more generally.

This illustration indicates that the distribution of vaccine effects among individuals is a major determinant of population-level impact and should be considered in evaluation. Specifically, the more homogeneously a vaccine acts, the lower its impact on disease transmission. Measures based on multipopulation study designs, spanning a range of transmission intensities, enable the inference of such distributions.

## Experimental Challenge

Infection in a controlled experimental setting is modeled by describing infected proportions in terms of challenge dose. Adopting standard formulations [15]–[17], the mean number of infecting pathogens is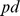, where *d* is the number of pathogens challenging the host, and *p* is the probability of infection for each pathogen; the number of infecting pathogens per host has a Poisson distribution with mean 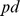. In the homogeneous case, the probability of a host remaining uninfected after pathogen challenge is the zero term of the distribution, leading to a probability of infection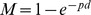, represented by the black curve in Figure 1B.

This model fails to fit many experimental data sets in which groups of hosts are exposed to varying doses of the pathogen, and the proportion infected in each group is calculated. In particular, the slope of the curve implied by this model is steeper than what is often observed. However, if individual hosts vary in their susceptibility to infection, a reduced slope arises. A simple model [11] assumes that the probability of each particle causing infection varies among hosts according to a beta distribution 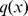, akin to the vaccine protection factor above, resulting in the modified dose-response 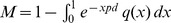.

Figure 1B illustrates dose-infectivity curves expected from an experiment in which groups of naive and vaccinated hosts are challenged with a range of pathogen doses under the distributions of protection described above, uncovering again a lack of uniform convergence to the all-or-nothing formulation.

We have adopted the same notation, 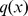, for susceptibility distributions in both natural transmission and experimental challenge settings to indicate the linkage between two arms of a unified study, as advocated here.

## Classification of Intervention Effects

**Figure 2:**
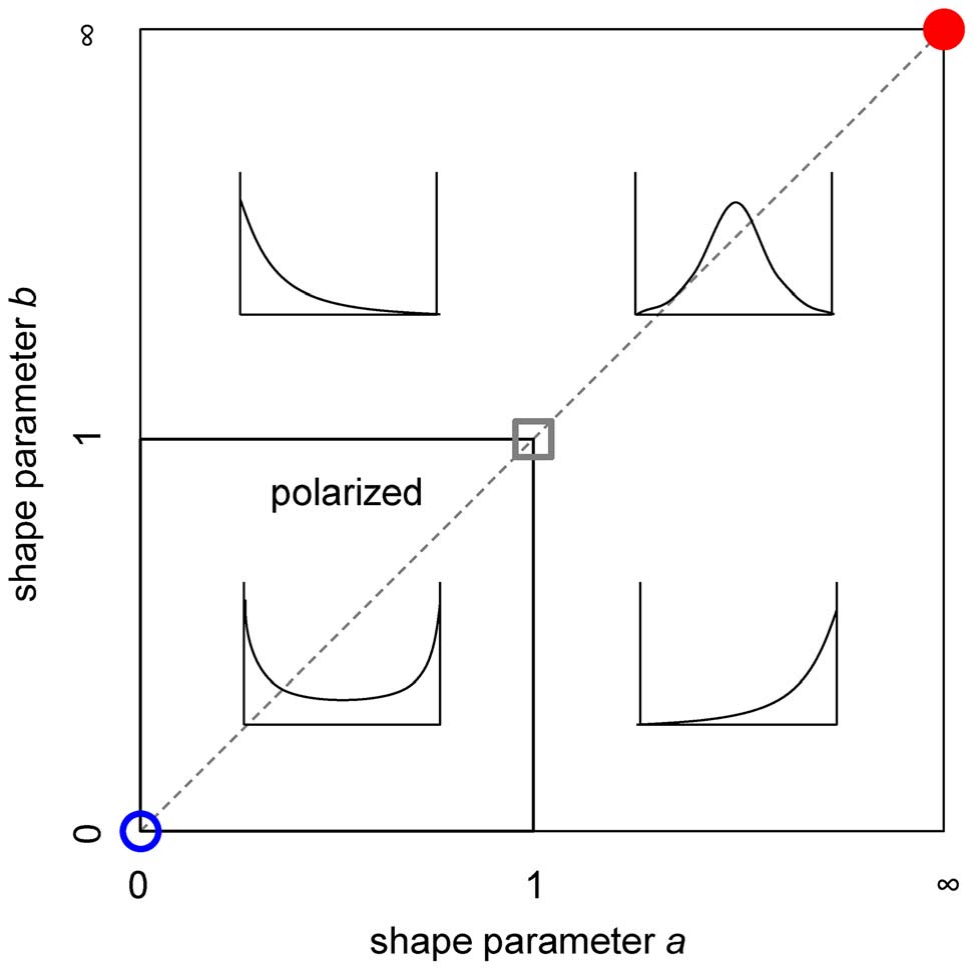
Shape classification in the terms of parameters *a* and *b*. Beta distributions are classified as: polarized if 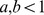; symmetric if 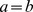 (gray dashed line), as in Figure 1; homogeneous in the limit 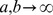 (red circle), as red in Figure 1; all-or-nothing in the limit 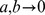 (blue circle), as blue in Figure 1; and uniform if 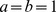 (gray square). The power to identify polarized distributions is analyzed in a neighborhood of the uniform distribution (Figure S1).

Experimental dose-infectivity curves provide information to infer the mode of action of interventions, such as vaccines. Given the lack of uniform convergence to all-or-nothing as the leaky mode becomes increasingly polarized, we have classified beta distribution shapes according to polarization (Figure 2). The dashed line along the diagonal indicates the location of the symmetric distributions used in Figure 1, and the circles indicate the location of the extreme homogeneous (red) and all-or-nothing (blue) distributions. The power to identify polarized distributions is analyzed in Figure S1, focusing on a vicinity of the uniform shape (gray square), showing good discriminatory power in the region of parameter space where uncertainty is greatest. This analysis suggests a promising approach for classifying intervention effects in controlled experimental settings and using this as prior information for further study in natural settings [18].

## Supporting Information

Figure S1Power analysis to identify polarized intervention effects. Simulated sets of dose-infectivity data were generated and used to estimate model parameters (Figure S2), assuming the host susceptibility of the intervention group described by a beta distribution, 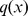, with shape parameters positioned as a grid in a square neighborhood of the uniform distribution, 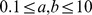. The procedure was applied 100 times to each of 1,600 grip points, and the number of correct shape classifications into polarized (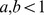) versus non-polarized (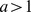 or 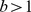) is represented. With 50 hosts per dose, the shape was identified with 95% accuracy in 57% of the simulated parameter space.(TIFF)

Figure S2Simulation and estimation experiment. A simulated set of dose-infectivity data was generated using models 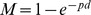 and 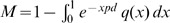 for nonintervention and intervention groups, respectively, where *d* is the dose (simulated at 10^4^, 10^5^, 10^6^, 10^7^, 10^8^, 10^9^, and 10^10^) and *p* is the probability of infection for each pathogen (simulated at 10^−6^). The host susceptibility of the intervention group is described by a beta distribution, 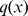, with shape parameters 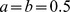. By fitting the models to the simulated data by a least squares procedure, we have estimated 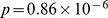, 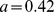, and 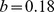. (A) The nonintervention arm of the experiment in black and the intervention arm in green. (B) Intervention effects' assumed distribution, shown as a dashed line, and the estimated distribution, represented as an unbroken line. This is an example in which a polarized intervention effect was estimated correctly.(TIFF)
