# Supplementary figures and images for: A Missing Dimension in Measures of Vaccination Impacts
Source: PLoS Pathog. 2014 Mar 6;10(3):e1003849. doi: 10.1371/journal.ppat.1003849 (PMC3946326; doi:10.1371/journal.ppat.1003849)

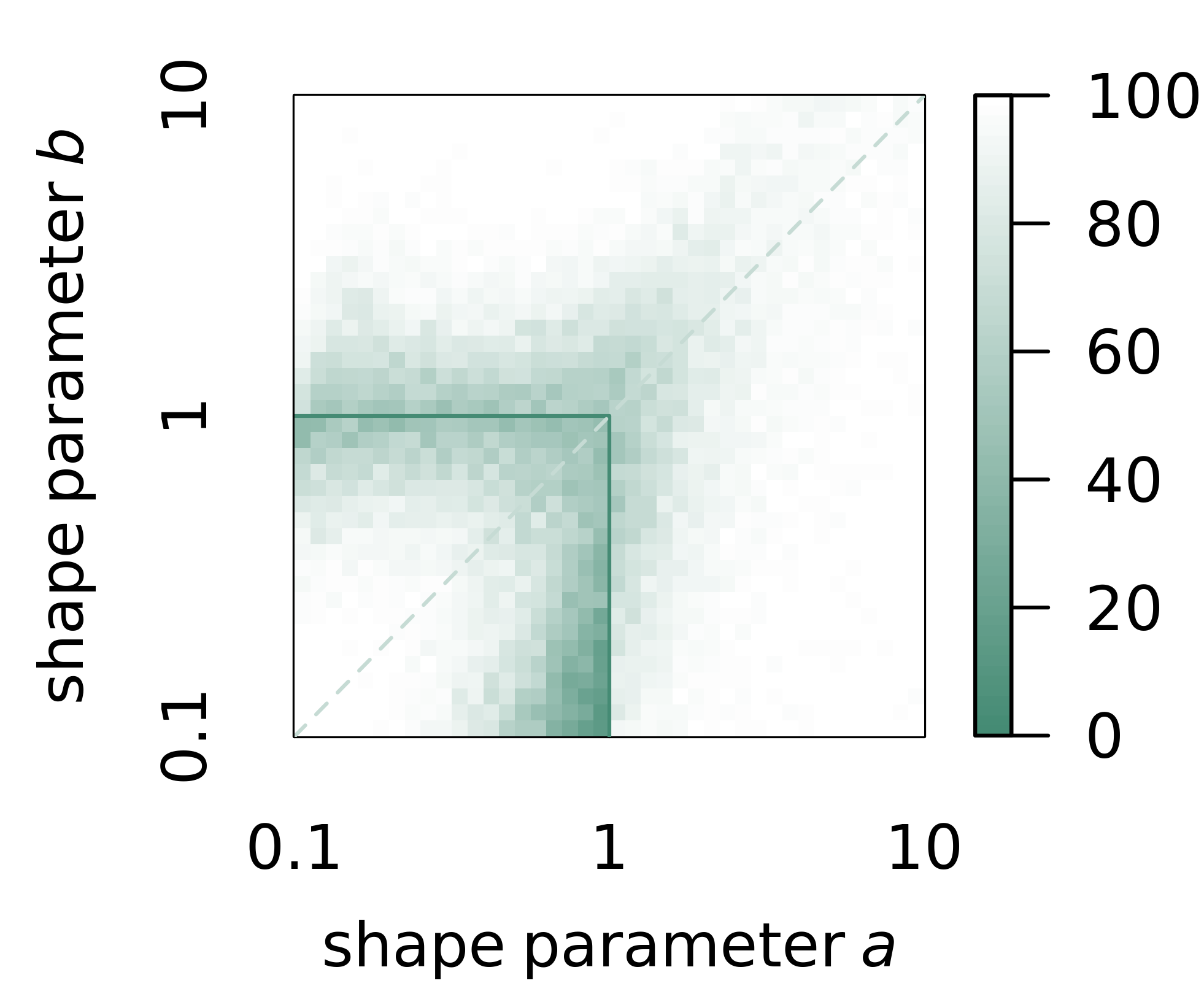

Supplement: Figure S1 — Power analysis to identify polarized intervention effects. Simulated sets of dose-infectivity data were generated and used to estimate model parameters (Figure S2), assuming the host susceptibility of the intervention group described by a beta distribution, , with shape parameters positioned as a grid in a square neighborhood of the uniform distribution, . The procedure was applied 100 times to each of 1,600 grip points, and the number of correct shape classifications into polarized () versus non-polarized ( or ) is represented. With 50 hosts per dose, the shape was identified with 95% accuracy in 57% of the simulated parameter space. (TIFF) [file ppat.1003849.s001.tiff]

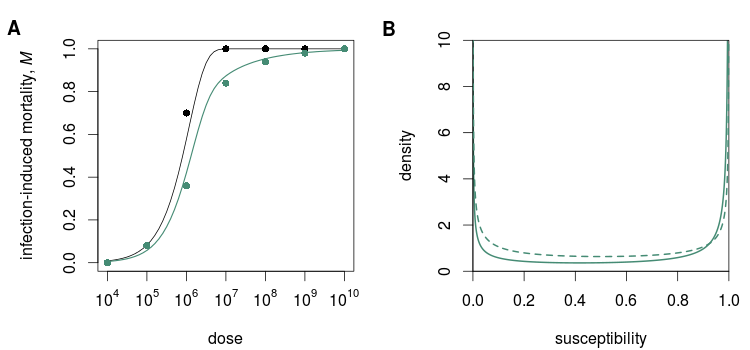

Supplement: Figure S2 — Simulation and estimation experiment. A simulated set of dose-infectivity data was generated using models and for nonintervention and intervention groups, respectively, where d is the dose (simulated at 104, 105, 106, 107, 108, 109, and 1010) and p is the probability of infection for each pathogen (simulated at 10−6). The host susceptibility of the intervention group is described by a beta distribution, , with shape parameters . By fitting the models to the simulated data by a least squares procedure, we have estimated , , and . (A) The nonintervention arm of the experiment in black and the intervention arm in green. (B) Intervention effects' assumed distribution, shown as a dashed line, and the estimated distribution, represented as an unbroken line. This is an example in which a polarized intervention effect was estimated correctly. (TIFF) [file ppat.1003849.s002.tiff]
